# Rheumatic disease and artistic creativity

**DOI:** 10.31138/mjr.30.2.103

**Published:** 2019-06-29

**Authors:** Katerina Chatzidionysiou

**Affiliations:** 1^st^ Department of Propaedeutic and Internal Medicine, Laiko Hospital, Medical School, National and Kapodistrian University of Athens, Athens, Greece

**Keywords:** Rheumatic disease, creativity, art

## INTRODUCTION

**Figure 1.**
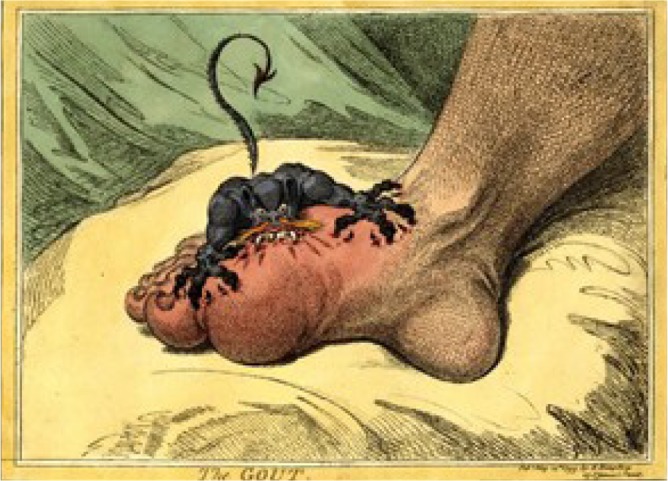
“*The Gout”*, Hames Gillray, 1799.

**Figure 2.**
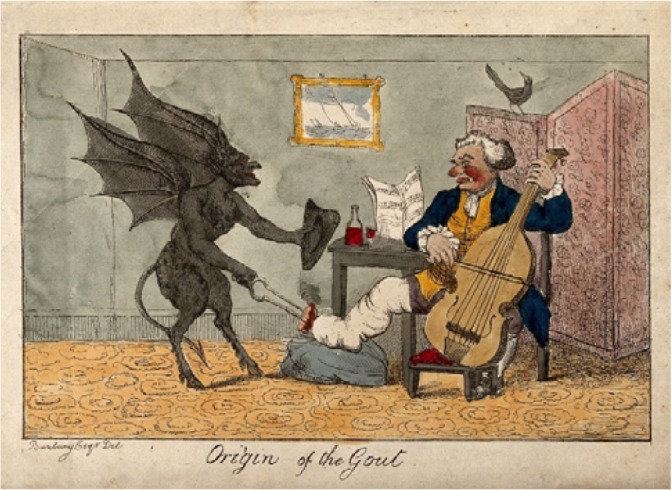
“*A gouty man who is drinking wine and playing the cello”,* H. W. Bunbury, 1785.

**Figure 3.**
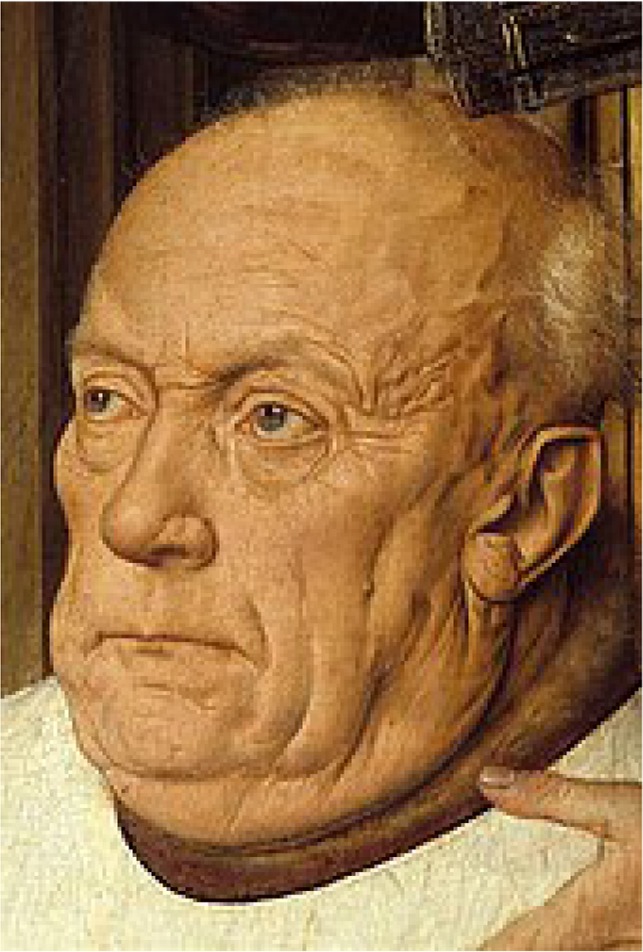
Detail from “*The Virgin and Child with Canon van der Paele”* by Jan van Eyck, 1434–36. We can observe the prominent temporal artery suggestive of arteritis.

**Figure 4.**
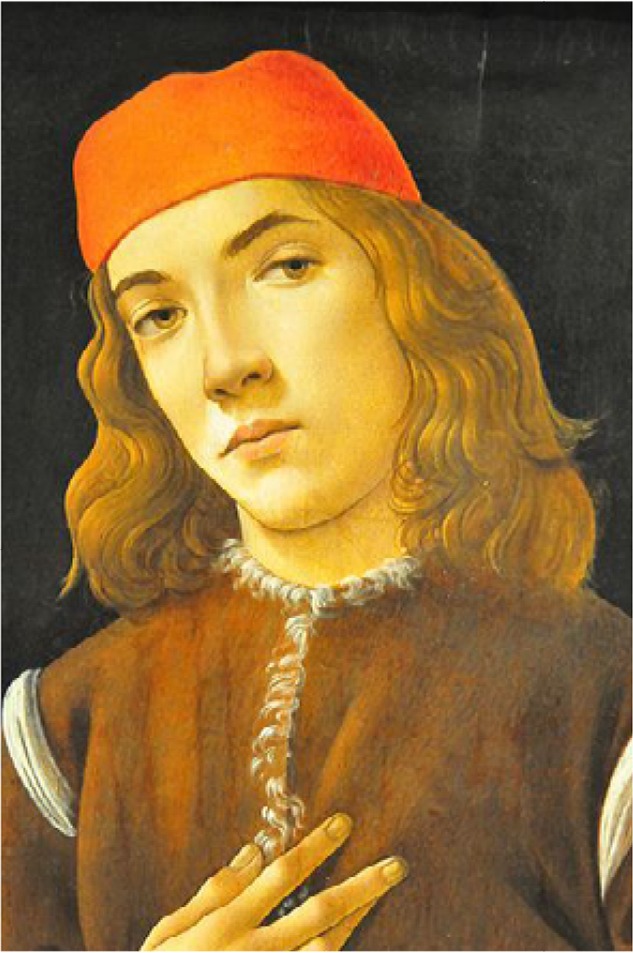
“*Portrait of a Youth”* by Sandro Boticelli, 1483. The changes depicted in his right hand with swelling and deformity of the fifth digit has been interpreted as arthritis, possibly juvenile.

**Figure 5.**
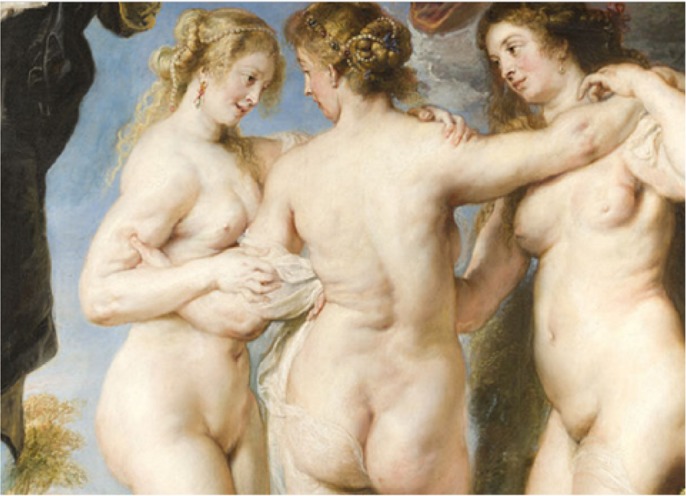
“*The Three Graces”*, by Rubens, 1630–1635. The changes depicted in the right hand of the Grace in the left of the painting could be attributed to rheumatoid arthritis.

Disease in general, and especially rheumatic disease, has inspired artists for centuries and has often been depicted in paintings.^1^ There are several examples of that; *“The Gout”*, by James Gillray in 1799, depicts a bare foot with classical signs of inflammation in gout attack such as extreme swelling, redness and severe pain presented as a demon biting the foot (*Figure 1*);^2^ “*A gouty man who is drinking wine and playing the cello”* by H. W. Bunbury is another excellent depiction of the excruciating pain and burning (also represented by a demon here) caused by the deposition of natrium urate crystals in the joints of patients with gout, and also gives important information about the close connection between gout and alcohol, one of the most common risk factors for gout (*Figure 2*); in “*The Virgin and Child with Canon van der Paele”* by the Early Netherlandish painter Jan van Eyck, Canon van der Paele is depicted with a prominent, swollen left temporal artery, suggestive of temporal arteritis (*Figure 3*);^3^ in Sandro Boticelli’s *“Portrait of a Youth”*, we can observe swelling of the wrists and PIP joints, as well as deformity of the fifth digit of the hand, that could be attributed to arthritis (*Figure 4*).^4^ However, some authors claim that this is a misinterpretation, and the way he painted hands was a stylistic configuration seen in other paintings as well;^5^ in “*The Three Graces”* by Rubens, we can observe deformities suggestive of rheumatoid arthritis (RA) (*Figure 5*). This suggests not only the presence of RA at that time, but also that Rubens himself might suffered from the disease.^6^ We can learn a lot about the natural history of rheumatic diseases through paintings, about the characteristics and the course of the disease at different periods of time.

## RHEUMATIC DISEASE AND ITS INFLUENCE IN ARTISTIC CREATION

Some authors claim that the influence of illness on artistic creation is little, and most of the associations often made are merely speculations. However, if we take into consideration that art is the supreme way of expressing our innermost feelings, as happiness, fear, despair, pain, both physical and mental, there are some indisputable examples of the close relationship between disease and artistic work. Apart from the obvious influence of disease in painter’s art in expressing their feelings relating to the disease, rheumatological conditions have often played a crucial role in the artistic style of many painters, and in some cases, it was the change of the style due to the disease that led to the creation of some of the greatest masterpieces of the artists.

**Figure 6.**
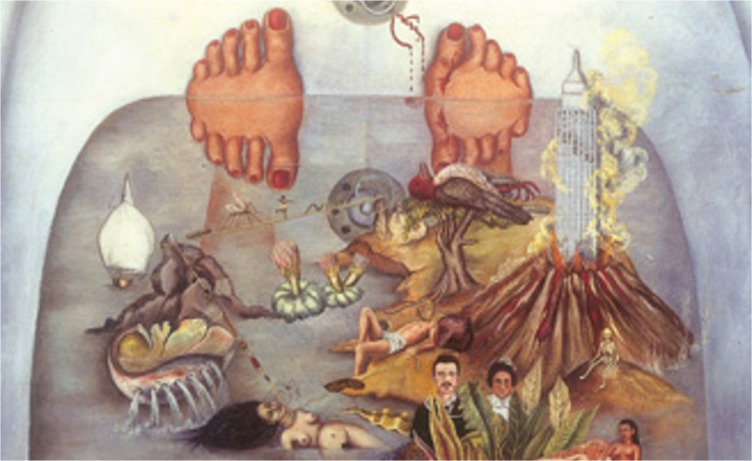
“*What the water gave me”* by Frida Kahlo, 1938. The sore between her toes are typical lesions of the congenital defect of spina bifida.

Frida Kahlo (1907–1954) was a famous Mexican painter and a feministic icon, famous for depicting paintings her increasingly painful congenital disorder in her. Although it is often wrongly considered that her suffering was due to injuries suffered when a streetcar plowed into a bus she was riding when she was 18, the main cause of her suffering was a congenital malformation, spina bifida.^7^ This was the cause of her progressive trophic ulcers on her lower extremities (*Figure 6*). Most of her paintings deal with pain, despair and death: as she herself stated, “my painting carries with it the message of pain”, and thus her art is closely related to chronic pain.

**Figure 7.**
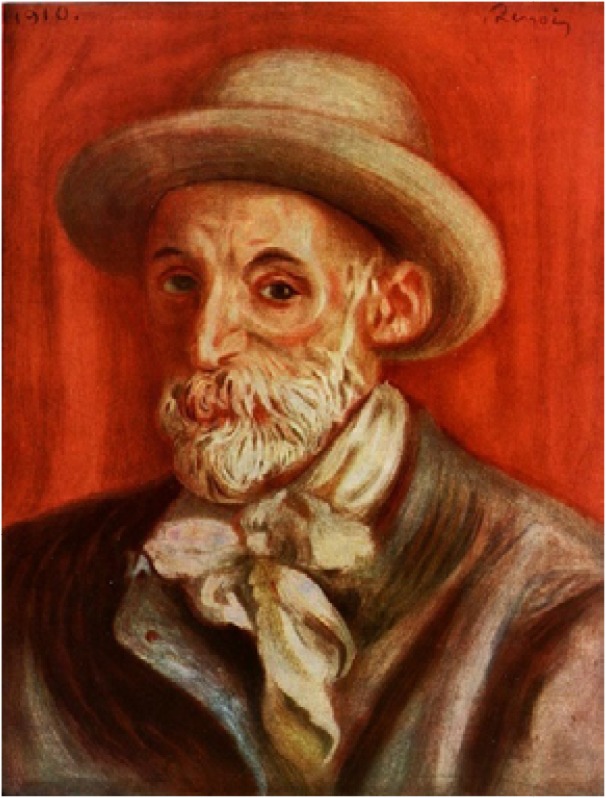
Self-portrait, Renoir, 1910. Cachexia is obvious.

Pierre-Auguste Renoir (1814–1919), the famous French impressionist, suffered from severe RA the last 25 years of his life.^8^ Living in a time before the emergence of the potent disease-modifying drugs that are available today, he developed severe deformities and loss of function of his joints. His right shoulder was ankylosed, as were his hands. He also developed several extra-articular manifestations, pleuritis, vasculitis with sores and at the end gangrene, nodules, rheumatoid cachexia, the latter seen in his self-portrait (*Figure 7*). He also suffered a stroke. By the end he was wheelchair-bound, which could have been due to post-stroke hemiplegia, but some authors claim that it was due to neurological complications from his RA.^8^ He died in 1919 of pneumonia.

**Figure 8.**
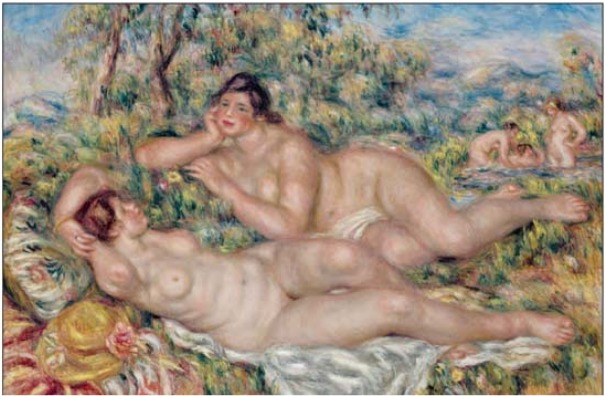
“*Les Grandes Baigneuses”*, by Renoir, 1918–1919, one of his paintings that was created with the “moving canvas” technique.

Remarkably for that time, Renoir created his own exercises, played the piano and ball games as a way to retain as much functionality of the joints as possible. He also developed many ways in order to continue creating art. He used to fix his palette on the arm of his wheelchair, attached the brushes to his hands with bandages and used a moving canvas or picture roll in order to counter-poise the lack of ability to move his arm and stand up.^8,9^ “*Les Grandes Baigneuses”* is one of his masterpieces painted in this way (*Figure 8*). His deformities and the ways he used to overcome his limited movement and continue painting unavoidably led to adaptions in his style. He started using small and rapid strokes of thinned paint, and he stopped mixing the colour on the canvas. These changes led to the creation of some of the greatest masterpieces of impressionism.

**Figure 9.**
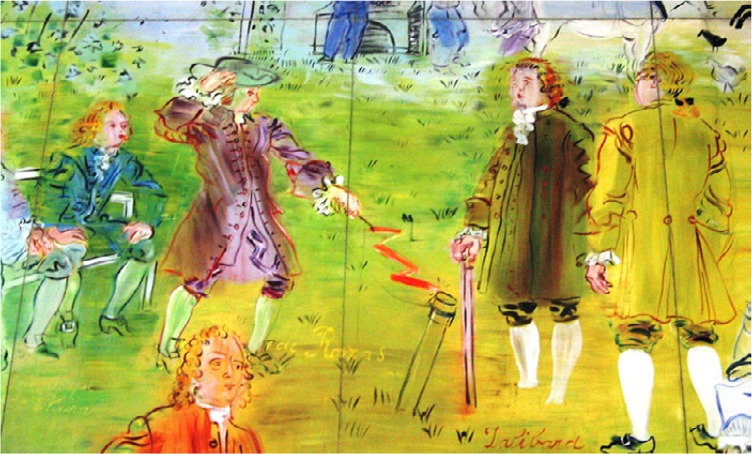
“La Fee Electricite”, Dufy, 1937.

Renoir was an extremely prolific painter, despite his severe illness. He used art as a way to cope with his pain. “Blessed painting. Even late in life, you are still creating illusions and occasionally giving joy,” he wrote to his friend, the painter Albert André.^10^ His bright colours he used to capture the beauty of real life and of humans without problems and the deification of nature, justify his title as “painter of happiness”. In contrast to Kahlo, Renoir gives us a different example of how suffering and pain can lead to the need to express the beauty of life; perhaps as a way of therapy. As Matisse witnessed: “A lengthy martyrdom – his fingers-joints were swollen and horribly distorted – yet he now painted his best works! While his body wasted away, his soul seemed to gain strength and he expressed himself with increasing ease”. Raoul Dufy (1877–1953) was a French fauvist also famous, as Renoir is, for his positive approach to life through his paintings, featuring happiness, luxury and pleasure. Gertrude Stein once admiringly said: “Raoul Dufy is pleasure itself”. He developed RA, his symptoms starting in the beginning of 1930. In 1937 he completed his masterpiece “*La Fee Electricite*”, a huge fresco for the International Exposition in Paris (*Figure 9*). Dufy included portraits of 110 famous scientists and thinkers who contributed to the invention and development of electricity, as well as elements of mythology and allegory. If we look closer, we can see a figure with swollen joints in his right hand, holding a cane, next to an electric lightning bolt. Could it be a symbolism of his own rheumatism? Rheumatic pain is sometimes described by patients as electricity, and the depiction of a rheumatic hand next to the bolt might not be by chance.

**Figure 10.**
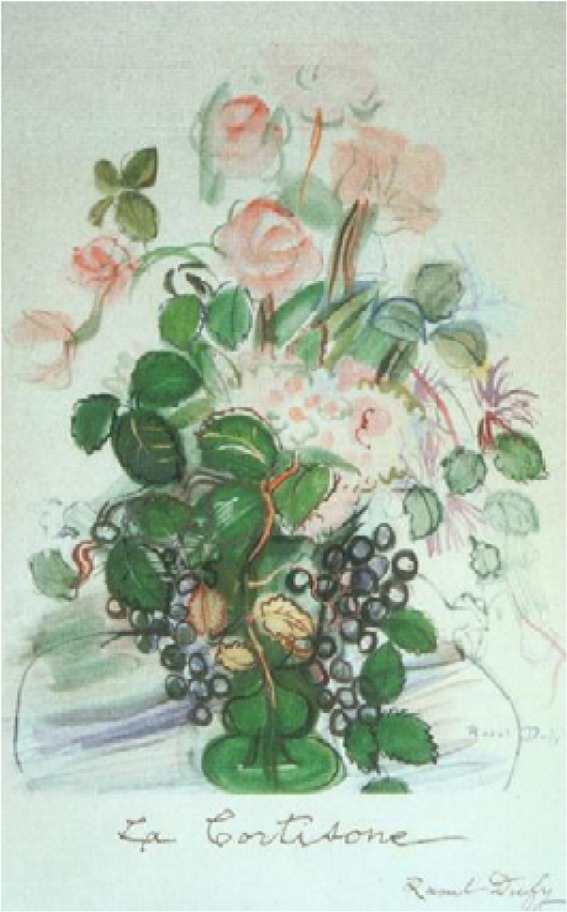
“*La Cortisone”*, Dufy, 1950.

It has been claimed that Dufy’s style changed as his disease progressed. The size of his paintings was reduced, the plots were less accurate, the hands were represented often distorted or sometimes were absent.^11^ Dufy was one of the first patients with RA to receive corticosteroids, after a personal invitation from Freddy Homburger, an oncologist and Professor of Medicine in Boston, who was also an amateur painter.^12^ At the time of his hospitalization, his RA was highly active and Dufy was almost completely immobilized. The result of the therapy was amazing, with Dufy becoming mobile within a few days and able to start physiotherapy. He regained his creativity for several more years. Among many other paintings, he painted a beautiful bouquet of anemones entitled “*La Cortisone*” (*Figure 10*). Interestingly, Renoirs’ last painting was an anemone. In 1953 Dufy died of an intestinal haemorrhage, possibly as a complication of the corticosteroids in combination with aspirin.^13^

Paul Klee (1879–1940) was a post-impressionist artist considered one of the most influential artists of the 20^th^ century.^14^ His highly individual style was influenced by several art movements, including expressionism, cubism and surrealism. He developed systemic sclerosis at the age of 57, with many typical manifestations of the disease, such as Raynaud’s phenomenon, scleroderma with flexion contractures and subsequently ulcerations, dysphagia, arthralgia, dyspnoea probably due to lung fibrosis or pulmonary hypertension, and ultimately heart failure. He finally died 4 years after diagnosis.^15^

**Figure 11.**
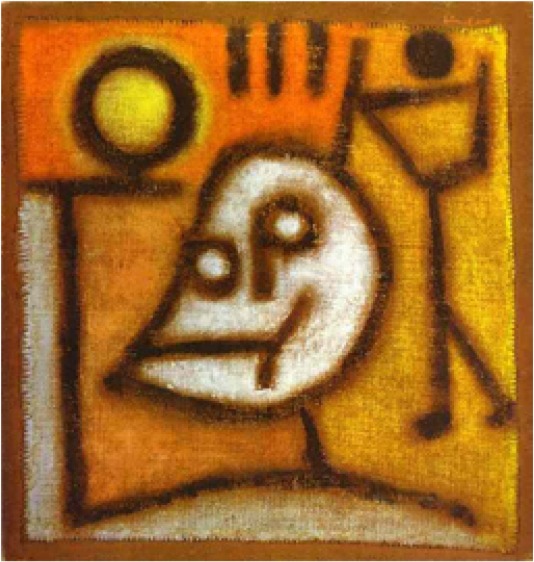
“*Death and fire*”, Klee, 1940, one of Klee’s last paintings.

**Figure 12.**
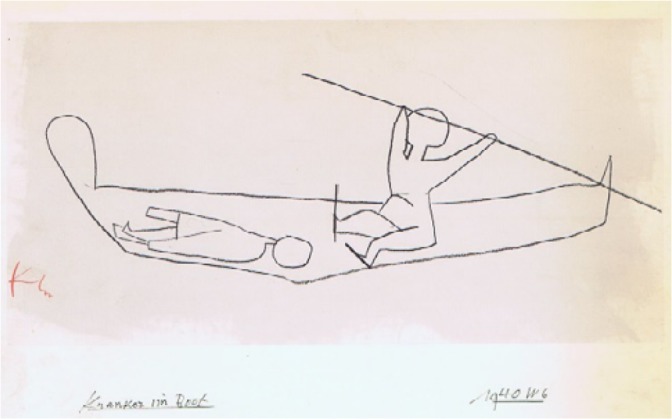
“*The sick one in the boat”,* Klee, 1940. Another example of how his illness affected his art, both his style and his thematology.

**Figure 13.**
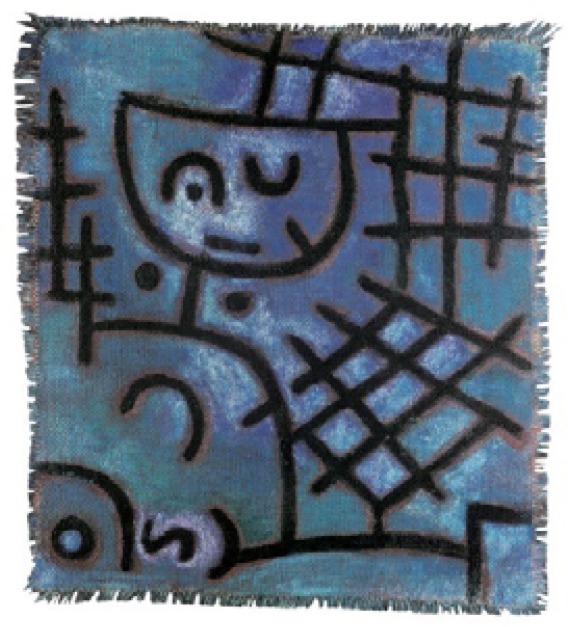
“*Captive”,* Klee, 1940. The prison might symbolise his hopelessness, fear, loneliness and entrapment due to his disease.

**Figure 14.**
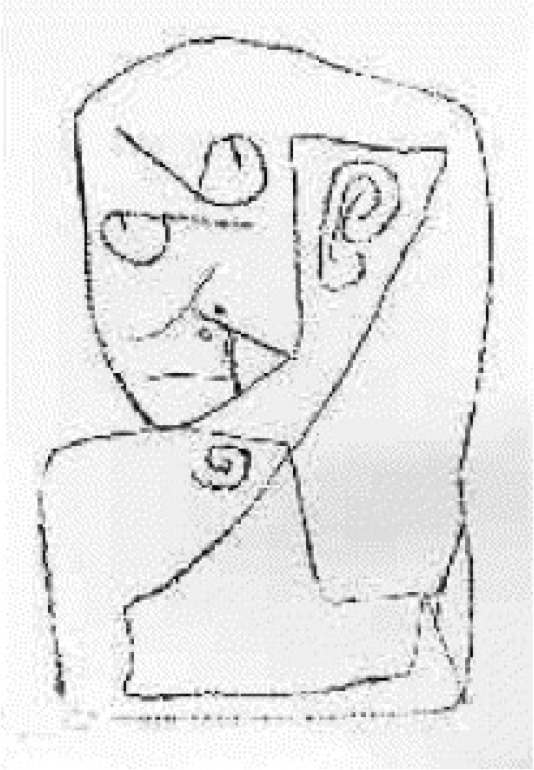
“*Endure!”,* Klee, 1940. Possibly his last painting. The resemblance with one of his photos from his last years is striking, showing the changes his systemic sclerosis made on his image.

Klee’s style changed profoundly as a consequence of his disease. His productivity declined at the beginning, but one year before his death, he created more than 1000 works of art. Intricate, small-scale compositions gave way to larger and coarser pieces. However, while at the beginning he used bright colours, after his diagnosis, his style is characterized by broad brush strokes, heavy, black crayon-like lines and dull colours.^15^ Many of the paintings he created during the last period of his life exemplify the impact his illness had to the theme of his art, depicting dysmorphia, pain, suffering and fear of upcoming death:^16^ “*Death and fire*” (*Figure 11*), “*The sick one in the boat*” (Figure 12), “*The mask*”, “*Repair*”, “*A sick man making plans*”, “*Captive*” (*Figure 13*). The drawing, “*Endure*!” (*Figure 14*), maybe his last drawing, is another example of how much impact his disease had on him, showing his disfigured face.

Going back in time, we will examine the case of another great master, the Spanish painter Francisco de Goya (1746–1828). At age 46, Goya suffered from a severe illness that lasted several months. It was characterized by loss of vision and hearing, as well as neuropsychiatric symptoms such as severe headaches, hallucinations and confusion.^17^ After a few months he recuperated, but was left deaf forever. He suffered a milder episode with similar symptoms two years before, suggesting a relapsing condition.^4^ In addition to the physical effects, his emotional health was affected. The precise cause of this illness has long been debated. One early yet unlikely hypothesis was that he had syphilis.^18^ Another possible diagnosis could have been a rare syndrome called Vogt-Koyanagi-Harada or uveomeningoencephalytic syndrome. This is an autoimmune disorder which involves the visual and cochlear pigment derived from the neural crest.^19^ Lead poisoning could also explain the clinical picture, at least partially. However, the relapsing nature of the disease, the regression of symptoms without therapy and the classic triad of encephalopathy, branch retinal artery occlusions and hearing loss are suggestive of Susac’s syndrome, a rare primary vasculitis of the central neural system.^20^

**Figure 15.**
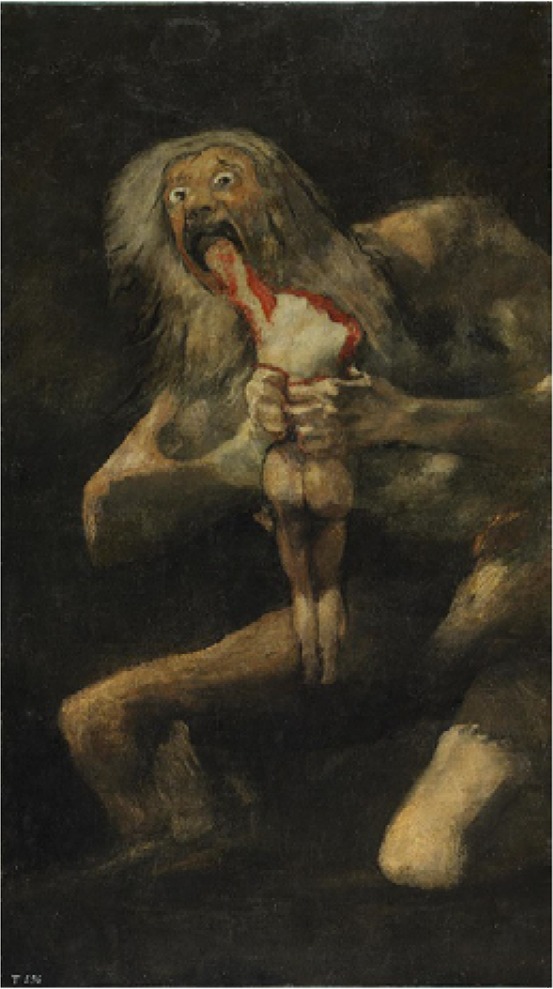
“*Saturn Devouring His Son”,* Goya, 1819–1823.

**Figure 16.**
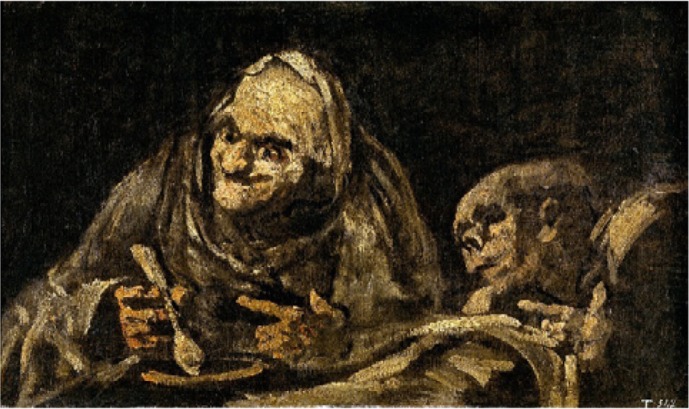
“*Two old men eating soup”,* Goya, 1819–1823.

Goya started his career in Madrid’s Royal Tapestry and later as a painter to the King, in 1786, creating portraits very “mainstream” for that time. In 1790 he became ill for the first time, and 2 years later, he had a second attack with the symptoms described above. He became isolated because of his permanent deafness. Perhaps as a result of his disease, he started expressing disaster, chaos, fear and terror in his works after recovery. His paintings became dark and macabre. An example of this is his famous black paintings, a collection of fourteen, horrifying paintings including “*Saturn Devouring His Son*” (*Figure 15*), “*Two old men eating soup*” (*Figure 16*), “*Men Reading*”, and others.

It is unquestionable that Goya became known due to his later works with a unique style, when he explored ideas of naturalism, fantasy and self-expression.^21^ This is highly possible that it was trigger by his illness. Thus, the “first of the moderns” might not have reached the same levels of creation without his terrible disease.

## CONCLUSION

There is a close relationship between disease and art. Rheumatic disease has inspired many painters as a theme, but most importantly it has deeply influenced painters who suffered from diverse rheumatological conditions, to express their suffering, pain, despair, but also hope, through their art. It could therefore be considered an excellent, complex and complete patient-reported outcome, and at the same time a therapeutic method. More interestingly though, from an artistic perspective, and almost ironically, the disease was in many cases the force that led to change in style and technique that contributed substantially to their establishment as great masters, supporting the fact that some of the greatest, most beautiful art is born of great suffering.
